# Complete Genome Sequences of *Polynucleobacter* sp. Subcluster PnecA Strains SHI2 and SHI8, Isolated from an Oligotrophic-Dystrophic Lake in Japan

**DOI:** 10.1128/mra.00030-23

**Published:** 2023-03-01

**Authors:** Yusuke Ogata, Keiji Watanabe, Shusuke Takemine, Kaoru Kaida, Maki Tanokura, Wataru Suda

**Affiliations:** a RIKEN Center for Integrative Medical Sciences, Yokohama, Kanagawa, Japan; b Center for Environmental Science in Saitama, Kazo, Saitama, Japan; University of Southern California

## Abstract

Members of the genus *Polynucleobacter* belonging to the subcluster PnecA comprise freshwater bacterioplankton with worldwide distribution. Here, we report the complete genome sequences of two *Polynucleobacter* sp. strains (PnecA), SHI2 and SHI8, isolated from the surface water of an oligotrophic-dystrophic lake in a humid continental climate in Japan.

## ANNOUNCEMENT

Free-living freshwater bacterioplankton of the genus *Polynucleobacter* belonging to subcluster PnecA are distributed worldwide (1, 2). To date, only two species (Polynucleobacter rarus and Polynucleobacter kasalickyi) have been described (3, 4). Isolation and cultivation of PnecA bacteria have rarely been successful (3–6). Thus, the genomic information on PnecA bacteria is very limited. Therefore, accessing genomic information for PnecA bacteria is important for providing physiochemical and ecological insights into the taxon.

Here, we report the whole-genome sequences of two *Polynucleobacter* sp. subcluster PnecA strains, SHI2 (=JCM 18219 = NBRC 107834) and SHI8, which were isolated from a surface water sample (depth, 0 to 50 cm) collected from Lake Shirakoma, an oligotrophic-dystrophic lake in the humid continental climate of Minamisakugun (Nagano, Japan; 36°03′04.2″N, 138°21′42.0″E), on 4 July 2010. The 10-mL sample was filtered through a disposable syringe equipped with a 0.7-μm particle retention glass fiber filter (Whatman Puradisc 25 GF/F; Germany). After filtration, a 100-μL aliquot of filtrate was spread onto modified Reasoner’s 2A (MR2A) agar plates and incubated at 25°C for 2 to 7 days (7). A single bacterial colony for each strain was picked, inoculated into sterilized MR2A liquid medium (pH 7.2), and incubated at 25°C for 2 days with reciprocal shaking (120 rpm). The pure strain cell suspensions were preserved at −80°C as stocks in MR2A broth supplemented with 20% (wt/vol) glycerol. Cells from the glycerol stocks were inoculated and cultured in MR2A liquid medium, harvested by centrifugation, and used for genomic DNA extraction.

Genomic DNA was extracted from strains SHI2 and SHI8 using enzymatic lysis and phenol-chloroform-isoamyl alcohol extraction, as described previously (8). Whole-genome sequencing was performed using the Sequel II system (PacBio, USA). A library was prepared using the SMRTbell Express template preparation kit v2.0 (PacBio), with DNA shearing using a g-TUBE device (Covaris, LLC, USA) and a target length of 10 to 15 kb. The PacBio reads were converted to circular consensus sequencing (CCS) reads using CCS software v6.2.0 (https://github.com/PacificBiosciences/ccs) and assembled using the assembler Canu v2.1.1 with the parameters minReadLength = 2200 and minOverlapLength = 2200 (9). The generated contig was checked for circularization to remap the CCS reads using Minimap2 v2.24-r1122 (10). The genomic sequence obtained was annotated and rotated to start at *dnaA* using DFAST v1.2.18 (https://dfast.nig.ac.jp) (11). Default parameters were used for all software analysis unless otherwise specified. The reads obtained and genomic sequences generated are summarized in Table 1.

**TABLE 1:** Summarized read and contig data obtained for *Polynucleobacter* sp. (PnecA) strains SHI2 and SHI8

Parameter	Data for strain:	
	SHI2	SHI8
Data for quality-checked Sequel reads		
No. of reads	26,707	30,000
Total no. of bases	427,935,707	410,464,382
*N*_50_ (bp)	16,094	13,445
Total no. of contigs	1	1
No. of CDSs^*a*^	2,471	2,448
No. of rRNAs	3	3
No. of tRNAs	37	37
Coverage (×)	172	165
BioProject accession no.	PRJDB15085	PRJDB15085
BioSample accession no.	SAMD00571187	SAMD00571188
Sequence Read Archive (SRA) accession no.	DRR428553	DRR428554
Chromosome data		
Genome size (bp)	2,485,335	2,485,290
GC content (%)	40.6	40.6
GenBank/ENA/DDBJ accession no.	AP027203	AP027204

a CDSs, coding DNA sequences.

Based on the DFAST annotation results, the genome sequences of strains SHI2 and SHI8 contain genes predicted to encode polyphosphate kinase and kinase 2 (*ppk* and *ppk2*), which are associated with the intracellular accumulation of polyphosphate. Furthermore, these strains also contain genes encoding putative alanine dehydrogenase- and glycine dehydrogenase-like proteins associated with ammonification. Both strains lacked a putative rhodopsin-like protein-encoding gene.

### Data availability.

The genome sequences and raw data are available at GenBank/ENA/DDBJ under the BioProject accession number PRJDB15085. The BioSample, DDBJ Sequence Read Archive (DRA), and DDBJ accession numbers are provided in Table 1.
